# Examining Diurnal Differences in Multidisciplinary Care Teams at a Pediatric Trauma Center Using Electronic Health Record Data: Social Network Analysis

**DOI:** 10.2196/30351

**Published:** 2022-02-04

**Authors:** Ashimiyu Durojaiye, James Fackler, Nicolette McGeorge, Kristen Webster, Hadi Kharrazi, Ayse Gurses

**Affiliations:** 1 Armstrong Institute Center for Health Care Human Factors Johns Hopkins University Baltimore, MD United States; 2 Division of Pediatric Anesthesiology and Critical Care Medicine Department of Pediatrics Johns Hopkins University School of Medicine Baltimore, MD United States; 3 Division of Health Sciences Informatics Johns Hopkins University School of Medicine Baltimore, MD United States

**Keywords:** pediatric trauma, multidisciplinary health team, multi-team systems, social network analysis, electronic health record, process mining, fluid teams

## Abstract

**Background:**

The care of pediatric trauma patients is delivered by multidisciplinary care teams with high fluidity that may vary in composition and organization depending on the time of day.

**Objective:**

This study aims to identify and describe diurnal variations in multidisciplinary care teams taking care of pediatric trauma patients using social network analysis on electronic health record (EHR) data.

**Methods:**

Metadata of clinical activities were extracted from the EHR and processed into an event log, which was divided into 6 different event logs based on shift (day or night) and location (emergency department, pediatric intensive care unit, and floor). Social networks were constructed from each event log by creating an edge among the functional roles captured within a similar time interval during a shift. Overlapping communities were identified from the social networks. Day and night network structures for each care location were compared and validated via comparison with secondary analysis of qualitatively derived care team data, obtained through semistructured interviews; and member-checking interviews with clinicians.

**Results:**

There were 413 encounters in the 1-year study period, with 65.9% (272/413) and 34.1% (141/413) beginning during day and night shifts, respectively. A single community was identified at all locations during the day and in the pediatric intensive care unit at night, whereas multiple communities corresponding to individual specialty services were identified in the emergency department and on the floor at night. Members of the trauma service belonged to all communities, suggesting that they were responsible for care coordination. Health care professionals found the networks to be largely accurate representations of the composition of the care teams and the interactions among them.

**Conclusions:**

Social network analysis was successfully used on EHR data to identify and describe diurnal differences in the composition and organization of multidisciplinary care teams at a pediatric trauma center.

## Introduction

### Background

Multidisciplinary care teams in health care are increasingly being seen as a multi-team system (MTS) [1,2], where 2 or more teams communicate and coordinate to achieve overarching goals [3], such as providing optimal care. MTSs are different from traditional teams in that MTS constituent teams are interdependent, work across boundaries, share accountability, and function through a hierarchy of goals that determine how lower goals are accomplished to realize higher goals [3]. MTSs have three attributes as follows: (1) compositional attributes (eg, number of teams, size of teams, and changes in team composition), (2) linkage attributes (eg, interdependence, hierarchical structure, and communication structure), and (3) developmental attributes (eg, changes in team membership over time) [4], which support the specialization and flexibility that allow constituent teams to pursue lower goals while trying to achieve higher goals [5].

MTS are often seen in environments where tasks are ambiguous, multifaceted, dynamic, and urgent [5]. In health care, trauma teams that take care of patients with trauma are examples of MTS. The care of patients with trauma is complex, multidimensional, and time sensitive, requiring multidisciplinary collaboration among a variety of health care professionals (HCPs) with complementary expertise, [6] with high fluidity of team membership (ie, members join and others leave based on the needs of patients) [7]. In addition, staffing levels at trauma centers vary with the time of day and the day of week, such that services of HCPs deemed nonessential may not be available during *off hours* (nights and weekends) [8-11], necessitating changes and adaptation in MTS structures.

Assessment of MTS, as they perform their work in actual settings, is important to gain a better understanding of work as done (as opposed to work as imagined) [12] and to identify how to improve their performance given the *realities and variations of work* [1]. Social network analysis can enable the understanding and assessment of MTS at the compositional (ie, membership) and organizational (eg, subteam) levels [1,13]. Typically, such assessment is done through observation, which can be highly resource intensive and may not be practical to capture all the cognitive work of team members involved in the trauma. Moreover, a self-reported surveys [5], which relies exclusively on perceptions of care professionals may also be limited in its ability to provide rich details [3]. The ability to exploit *digital traces* [14], which may provide opportunities over survey data [15,16] or observational data, is desirable. Electronic health record (EHR) systems offer the opportunity to study the composition and organization of care teams working as part of an MTS [17]. EHRs capture many clinical activities that are performed by HCPs in the process of care delivery [17,18], and previous studies have shown the feasibility of obtaining plausible information about care teams from EHR data [17].

### Objective

This study aims to identify MTS and demonstrate the dynamic nature of the compositional and organizational structures of the MTS by describing diurnal differences at various locations in a pediatric trauma center using EHR data.

## Methods

### Research Setting

This study was conducted as part of a larger research project (AHRQ R01HS023837) [19-21] aimed at redesigning pediatric trauma work systems based on health information to improve care transitions and patient safety. This study builds on a core methodology that has been previously described and validated [22,23]. The core methodology is reproduced here from data set subsection to “generation of master event log” subsection with necessary modifications for this paper.

### Study Setting

This study was conducted at a large academic children’s medical hospital with a level I pediatric trauma center in the Eastern United States, which receives approximately 1000 pediatric patients with trauma per year. The participating hospital triages incoming patients into one of four trauma activation levels as follows: alpha (level I or highest severity), bravo (level II), critical trauma transfers (includes interfacility*,* but patients who are stable but critically injured, and is also known as a consult) and emergency department (ED) response, that are ordered by decreasing acuity and need for multidisciplinary care with ED response activations exclusively handled by the ED staff.

Trauma activation levels determine the composition of the trauma team, as specified by state [24] and institutional policy. The trauma team is derived from the ED staff, the general pediatric surgery service, pediatric intensive care unit (PICU), and the ancillary support staff (eg, child life specialists, chaplains, and social workers). Following resuscitation, if inpatient admission is required, patients with single-system injuries are admitted under the appropriate specialty service, whereas patients with multisystem injuries are admitted under the general pediatric surgery service, which is responsible for coordinating care among managing specialty services (eg, neurosurgery or orthopedic surgery).

The Johns Hopkins Medicine Institutional Review Board approved the study (IRB00076900).

### Data Set

Data were extracted from the pediatric trauma registry and the EHR data warehouse (ie, the Clarity database of Epic). We limited EHR data to encounters with trauma activation levels of alpha, bravo, and critical trauma transfers that were managed between January 1 and December 31, 2017. Demographic and encounter data including age, sex, origin of patient, trauma activation level, injury severity score, and Glasgow Coma Scale score were collected from the registry. Admission, discharge, and transfer (ADT) data and metadata of 5 clinical activities (ie, notes, procedure orders, medication orders, flow sheet entries, and medication administration entries) captured in the EHR were collected from the EHR data warehouse. For each EHR activity type, we obtained the encounter ID (visit ID), activity timestamp, unique ID, and generic clinical roles (eg, attending or resident) of the HCP that performed the activity. The note metadata included the service of the authors, whereas the procedure orders, medication orders, and medication administration entries included the care location (eg, ED or PICU) where the activity was performed.

### Data Preparation

Each encounter was assigned a randomly generated, unique study ID. Timestamps of EHR metadata were normalized by replacing them with time (in minutes) from ED arrival, which ensured that the temporal sequence of events was maintained for each encounter. Activities without a full complement of data were excluded. Activities that were initiated by the EHR system and initiated by student roles (eg, nursing and medical students) were also excluded as they bore no accountability for patient care. As notes were typically signed off much later from when they were started, we considered the note creation time as the note completion time. As flow sheet and note data lacked care location data, we inferred the care location for each activity from the ADT data as follows: First, a location timeline was generated from the ADT data (ie, sequence of admissions to various hospital locations from ED arrival to hospital discharge). The normalized timestamps of each activity in the flow sheet and note metadata were then subsequently related to the location timeline, and the corresponding care location was taken as the care location where the flow sheet and note activities were performed.

### Identification of Functional Roles

We considered collaboration at the level of functional roles (eg, ED nurse, neurosurgery resident, PICU fellow, and surgery attending) rather than individuals, as past studies have shown that mirrors the reality of clinical practice [25]. To determine functional roles, we identified the service (eg, orthopedic or ophthalmology service) to which each identified HCP belonged and prefixed it to their generic role (eg, resident or attending). This service could be a service that is bound to a care location (eg, ED, PICU, or general care floor) or a service that operates across care locations (eg, general pediatric surgery service or physical therapy).

We assumed that the services of certain functional roles (eg, attending, fellows, physician assistants, and nurse practitioners working on specialty services) were fixed as determined from their notes. Chart reviews and directory lookups were conducted to identify the services of individuals whose services could not be determined from the extracted metadata. The services of medical residents, which frequently change as they rotate through various services for their training, were determined on an encounter basis derived from the service of the attending that cosigned the notes. The services of registered nurses, unit-based nurse practitioners, and allied HCPs (excluding radiology technicians) were determined by taking the mode of the frequency distribution of the location of the activities they performed. The services of radiology technicians were determined on an encounter basis similar to that of residents.

Activities by individuals whose services could not be determined were excluded. Since the location of flow sheet and note activities were inferred, the records were excluded if the inferred location did not correspond to the base unit of HCPs.

### Methodologic Approach

We used a process mining approach, which is a field of data science that *aims to discover, monitor, and improve real processes by extracting knowledge from event logs* [26]. The starting point for process mining is an event log, which contains a collection of events. Each event represents a discrete activity (eg, note writing) in a given process (eg, clinical care), performed by an actor (eg, ED resident), and relates to a case (eg, patient encounter). Each event is time-stamped (eg, order placed on January 22, 2000, at 10:45 AM), allowing all events for a patient encounter to be ordered chronologically [27]. By applying a *metric* described below, social network interactions and collaboration between different functional roles were obtained [28].

*Working together* is a commonly used metric for representing collaboration in unstructured processes with frequent ad hoc behavior such as in health care [29]. The working together metric counts how frequently 2 actors work together on same cases [28]. In its regular form, the working together metric does not accommodate for temporal distance between actors, which is important in health care where different HCPs are involved in patient care at different stages of care. Consequently, we defined a variant of the working together metric, referred to as *working closely together*, to account for temporal distance among actors. The working closely together metric counts the number of times 2 actors worked closely together with respect to time for a given patient relative to the number of times the 2 actors had the opportunity to work together. To operationalize this metric, we considered the shift rotation as the unit of clinical work and collaboration, and assumed that functional roles that were involved in the care of a patient during a shift had the opportunity to work together, whereas functional roles that were captured in the EHR within a similar time interval were *working closely together*. Therefore, this metric translates to functional roles that are jointly involved in completing the same tasks or completing disparate tasks within the same time interval.

### Generation of Master Event Log

EHR metadata were processed into an event log consisting of the study ID, normalized time, EHR activity type, unique ID, and functional role of the HCP, and care location. Multiple *same-time events* were generated from notes, procedures, and medication orders that involved multiple HCPs. The encounter timeline was divided into shift rotations (day: 7 AM-6:59 PM and night: 7 PM-6:59 AM) numbered 0 to N, and each event in the event log was labeled with the corresponding shift number and shift type (day or night). Events within each shift were partitioned into segments based on *natural breaks* in the continuity of events. We assumed a natural break to be a minimum of 30 minutes between adjacent events in the event log to accommodate the lag between the occurrence of activities in real life and registration in the EHR. The Jenks Natural Break Optimization algorithm [30] was used to determine the optimal break interval between 30 and 120 minutes in 5-minute increments.

### Generation of Sublogs

The master event log was divided based on shift type (day or night) and care location (ED, floor, or PICU) to obtain six individual event logs: ED morning, ED night, floor morning, floor night, PICU morning, and PICU night.

### Network Representation

For each individual sublog, an undirected edge (ie, the relationship among nodes) was created for all pairwise combinations of identified functional roles within each event segment. Unique edges across all segments across all shifts across all encounters were obtained as the collaboration network. The weight of the edges was obtained by dividing the number of shifts an edge was present between 2 functional roles by the number of shifts in which both functional roles were involved, which effectively normalized the weights and accommodated for variation in care team composition across encounters.

### Threshold Selection

To prevent the capture of spurious edges (ie, edges that do not really exist or edges with spurious weights) in network analysis, a threshold number of shared encounters among nodes (ie, functional roles) is usually applied to constructed networks. The eventual network structure is sensitive to the selected threshold. Various approaches that have been used to determine this threshold are subjective [31], including arbitrary selection [32], clinician informed [33], and retaining only a fixed top percentage of the strongest edges [34]. In this study, we attempted to take a more objective approach to threshold determination by introducing a heuristic method akin to the elbow method [35], which is used to determine the optimal number of clusters in k-means clustering. For each event log, we obtained and plotted the rate of change of the total number of edges removed as the threshold value (ie, representing the number of shared shifts) was incrementally increased from 2 to 20 and obtained a LOWESS (Locally Weighted Scatterplot Smoothing)-smoothed curve of the plot. The elbow point—the smallest threshold value at which the rate of change becomes insignificant or constant, was taken as the optimal threshold. The underlying assumption of this method is that as the threshold of the shared number of encounters is increased, trivial and spurious edges are removed, and the network structure changes up to a point where further increases in threshold value result in minimal removal of edges with little or no change in the network structure. At this threshold point, we assume that the network structure is relatively stable and only significant edges and nodes remain.

### Network Visualization and Analysis

We used the igraph 1.1.1 package [36] in R (version 3.4.0; R Foundation for Statistical Computing) [37] to create and visualize the networks. From each network, we obtained the node count (ie, number of functional roles) and edge count (ie, number of relationships among functional roles). We used the linkcomm package 1.0.11 [38] to identify the overlapping communities in the networks. A community is a subnetwork that contains a high density of edges among members but fewer edges with members of the larger network, thus represents a tightly knit subgroup [39]. The linkcomm package is an R implementation of the algorithm by Ahn et al [40] that, as opposed to other community detection algorithms that cluster nodes—clusters edges assuming a node can belong to multiple communities, thus enabling the discovery of overlapping and nested communities. The algorithm by Ahn et al [40] is the most commonly used overlapping community detection algorithm and tends to produce superior performance if multiple ad hoc behaviors result in a high degree of overlap in derived networks, as is commonly seen in health care settings [41,42]. The algorithm uses a hierarchical clustering method to produce a dendrogram that, in the default setting, is cut at a level that maximizes the partition density [40]. The linkcomm package offers a unique visualization that uses different colors to depict edges and nodes that belong to different communities. Nodes are sized to reflect the number of communities the node belongs to, with larger nodes belonging to more communities. Nodes belonging to more than one community are also presented as pies with the pies divided and colored based on the proportion of the edges for that node in various communities that the node belongs. We parameterized the algorithm with the McQuitty hierarchical clustering method, also known as the Weighted Pair Group Method with Arithmetic Mean [43], so that edge weights can be considered in community determination. We subsequently obtained community-depicted networks produced at maximum modularity that were visualized with easily understandable network layout algorithms.

### Statistical Analysis

We obtained and compared descriptive statistics of demographic, injury, and outcome characteristics of day and night shift encounters. We also compared composition of days and night shift event logs for each care location. Differences among interval and categorical variables were examined using Wilcoxon rank-sum and Pearson chi-square tests, respectively. Differences were considered statistically significant at an α<.05. The analysis was performed using Stata 13 [44].

### Validation

Two forms of validation were conducted. In the first validation step, we compared the results of this study with the secondary analysis of data from and results of a previous study [45] in which we developed a *role-location matrix*, which is a 2×2 table of functional roles and the inpatient locations in which they typically worked via semistructured interviews with clinicians (n=21) and subject matter experts (n=22), and a review of the institutional and trauma registry protocol. We compared the functional roles and the locations in which the functional roles were found in this study to the role-location matrix. In the second validation step, we validated the collaboration patterns of pediatric trauma MTS via member-checking interviews (n=6) with care professionals (ie, pediatric trauma program director, PICU attending, and pediatric trauma nurses) that were involved in pediatric trauma care. The interviews were conducted by AD, KW, and GSD and APG as a group. During each session, the collaboration patterns of care teams were individually presented to the HCP, who were asked to comment on (1) the accuracy and completeness of the roles that were captured by location and shift; (2) whether the collaborative patterns mirrored reality or not; and (3) whether the differences between day and night patterns for a given care location (ie, ED, PICU, or floor) were suggestive of reality.

## Results

### Overview

There were 413 encounters in the cohort, of which 65.9% (272/413) and 34.1% (141/413) began during day and night shifts, respectively. Compared with patients who arrived during day shifts, those who arrived during night shifts were significantly older (median age 7 vs 10 years; *P*=.04), had a higher proportion of critical trauma transfers (8.8% vs 26.2%; *P*<.001), and had a higher proportion of penetrating injuries (5/272, 1.8% vs 11/141, 7.8%; *P*<.001; Table 1). There were no significant differences in sex, injury severity score, Glasgow Coma Scale, operating room and PICU admissions, ED, PICU, hospital length of stay, and mortality.

**Table 1:** Comparison of demographic and encounter characteristics by shift type^a^.

Variables		Day (n=272)	Night (n=141)	*P* value	
Age (years), median (IQR)		7 (3-11)	10 (3-13)	.04	
Male sex, n (%)		184 (67.7)	83 (58.9)	.08	
**Trauma activation, n (%)**					<.001
	Alpha	26 (9.6)	5 (3.6)		
	Bravo	222 (81.6)	99 (70.2)		
	Critical trauma transfer	24 (8.8)	37 (26.2)		
**Origin, n (%)**					<.001
	Scene of injury	245 (90.1)	102 (72.3)		
	Transfer	2 (0.7)	38 (27)		
	Others	2 (0.7)	1 (0.7)		
**Injury type, n (%)**					.01
	Blunt	259 (95.2)	126 (89.4)		
	Penetrating	5 (1.8)	11 (7.8)		
	Others	8 (2.9)	4 (2.8)		
ISS^b^, median (IQR)		5 (2-10)	5 (2-9)	.76	
GCS^c^, median (IQR)		15 (15-15)	15 (15-15)	.48	
ED^d^ LOS^e^ (minutes), median (IQR)		253.5 (187-361)	254 (146-374)	.52	
OR^f^ admission, n (%)		41 (15.1)	22 (15.6)	.89	
PICU^g^ admission, n (%)		43 (15.8)	27 (19.2)	.39	
PICU LOS (days), median (IQR)		1 (1-3)	1 (1-2)	.48	
Hospital LOS (hours), median (IQR)		7 (4-32)	14 (4-41)	.21	
Mortality, n (%)		7 (2.6)	2 (1.4)	.72	

^a^Day shift is defined as 7 AM to 6:59 PM, whereas night shift is defined as 7 PM to 6:59 AM.

^b^ISS: injury severity score.

^c^GCS: Glasgow Coma Scale.

^d^ED: emergency department.

^e^LOS: length of stay.

^f^OR: operating room.

^g^PICU: pediatric intensive care unit.

### Master Event Log Characteristics

There were 837,318 events in the initial event log, respectively. Only 0.19% (1564/837,318) of the events were excluded owing to the inability to resolve the functional role of the actor. Consequently, 835,754 events remained in the master event log. Flow sheet entries accounted for 89.45% (749,000/837,318) of all events in the log. A total of 1647 unique HCPs occupying 110 functional roles were identified, of which 58 functional roles were recorded in at least 4.8% (20/413) of encounters. The ED registered nurses were recorded in all 413 encounters, whereas the ED attending, ED resident, and ED radiology technician were recorded in 98.5% (407/413), 93.2% (385/413), and 80.6% (333/413) encounters, respectively.

### Comparison of Sublogs Obtained Based on Shift Type and Care Location

**Figure 1:**
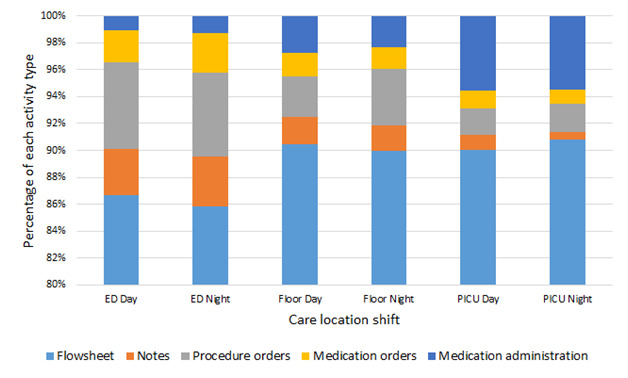
Comparison of the composition of various activity types by care location and shift type. ED: emergency department; PICU: pediatric intensive care unit.

Figure 1 depicts the composition of the individual sublogs for each care location and shift duty. The proportions of various activities in the day and night logs for each care location were similar, with some notable differences. The ED night log contained more medication administration orders than the ED day log, which contained more flow sheet events. The floor day log contained more medication administration than the floor night, which contained more procedure-order events. The PICU day contained more notes events that the PICU night, which contained more flow sheet events.

### Threshold Selection

**Figure 2:**
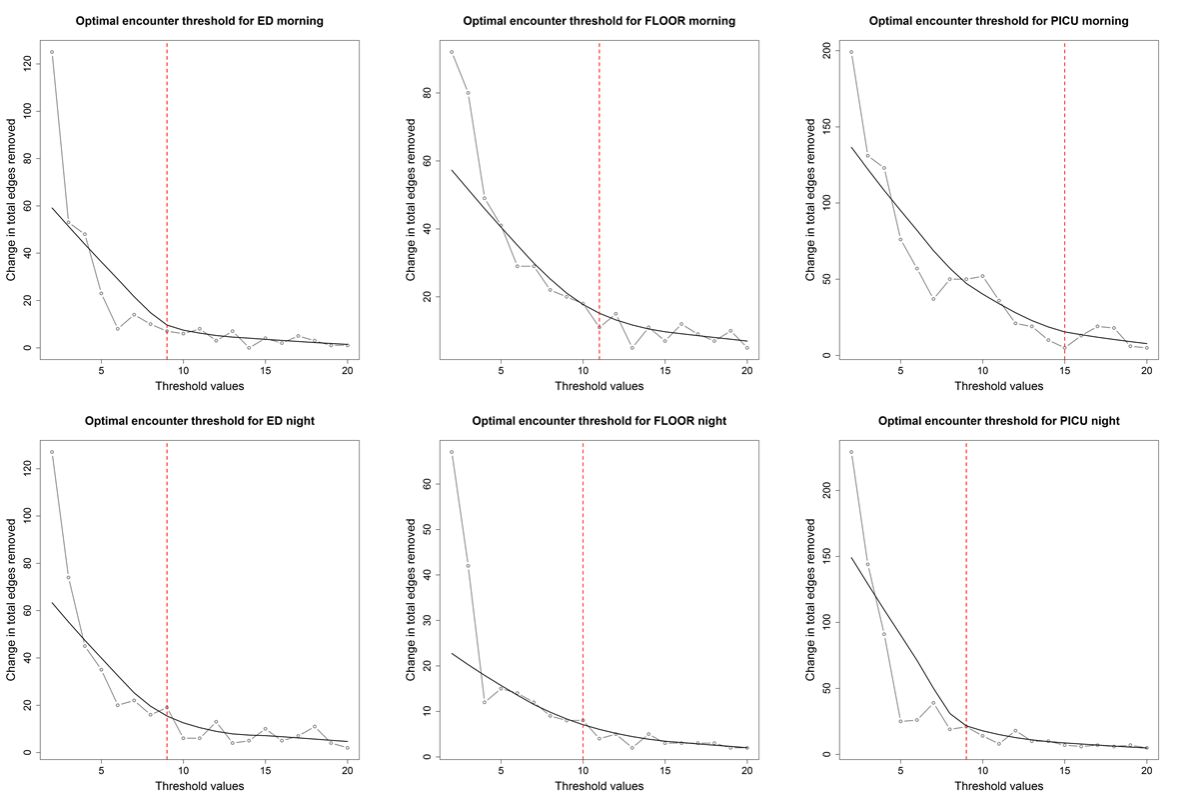
Determination of encounter threshold for each event log. ED: emergency department; PICU: pediatric intensive care unit.

Figure 2 shows the plots of the rate of change of total edges removed against increasing threshold values. The gray line and point plot show the difference in edges removed as the threshold is increased, whereas the smooth black line is the LOWESS curve. Some LOWESS curves, such as the ED morning and PICU night, have sharply defined elbows, whereas others have subtle elbows. The red vertical lines indicate the selected threshold number of shared encounters by HCPs for each event log. For both ED day and night, the threshold was determined to be 9. For the floor, 11 and 10 were selected as the thresholds for day and night, respectively, whereas for the PICU, 15 and 9 were selected as the day and night thresholds, respectively.

### Collaborative Care Teams in the Pediatric ED

**Figure 3:**
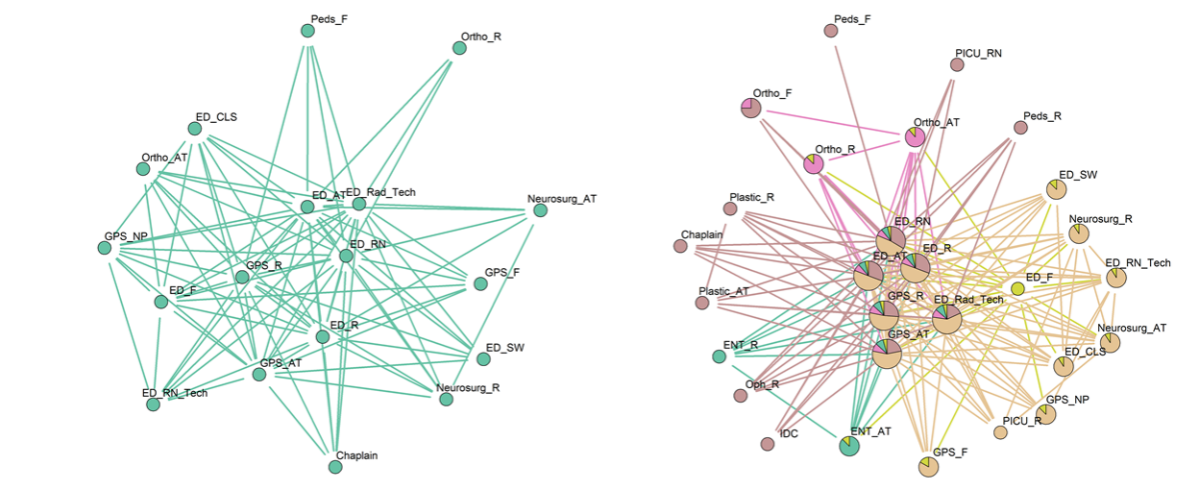
Collaborative care team patterns in the emergency department. Left: day shift; right: night shift.

Figure 3 shows the collaborative care team pattern in the ED during the day and at night visualized using the Kamada Kawai layout algorithm [46], which is a force-directed algorithm. Table 2 contains the meaning of the abbreviations used in Figure 3 and in all other network diagrams in this paper. The day pattern contained 18 nodes and 87 edges, whereas the night pattern contained 28 nodes and 160 edges. The night pattern was distinctively star-shaped and had 5 overlapping communities with the ED attending, residents, nurses, radiology technicians, and the general pediatric surgery attending and resident forming the core and belonging to all 5 communities. The day pattern had a less distinctively defined star pattern and had only 1 community. Attending-resident pairs from neurosurgery and orthopedic surgery services, and allied HCPs, including social workers, chaplains, and child life specialists, were at the periphery in both patterns. Attending-resident pairs from otolaryngology and plastic surgery were seen only in the night pattern and belonged to separate communities, whereas only the resident from the ophthalmology service was seen in the night pattern. The PICU nurse, resident, and the imaging data coordinator (IDC) were also seen in the night pattern.

**Table 2:** Abbreviations used in the network diagrams.

Abbreviation	Meaning
Anes	Anesthesia
AT	Attending
CLS	Child life specialist
CM	Case manager
DT	Dietitian
ED	Emergency department
F	Fellow
GPS	General pediatric surgery
HCC	Home care coordinator
IDC	Imaging data coordinator
Neuro	Neurology
Neurosurg	Neurosurgery
NP	Nurse Practitioner
Oph	Ophthalmology
Ortho	Orthopedic surgery
OT	Occupational therapy
PA	Physician assistant
Peds	Pediatrics
Pharm	Pharmacist
PICU	Pediatric intensive care unit
PMR	Physical medicine and rehabilitation
PPS	Pediatric pain service
PT	Physical therapist
R	Resident
Rad_Tech	Radiology technician
RN	Registered nurse
RN_Tech	Nurse technician
SW	Social work

### Collaboration Patterns of Care Teams in the Floor

**Figure 4:**
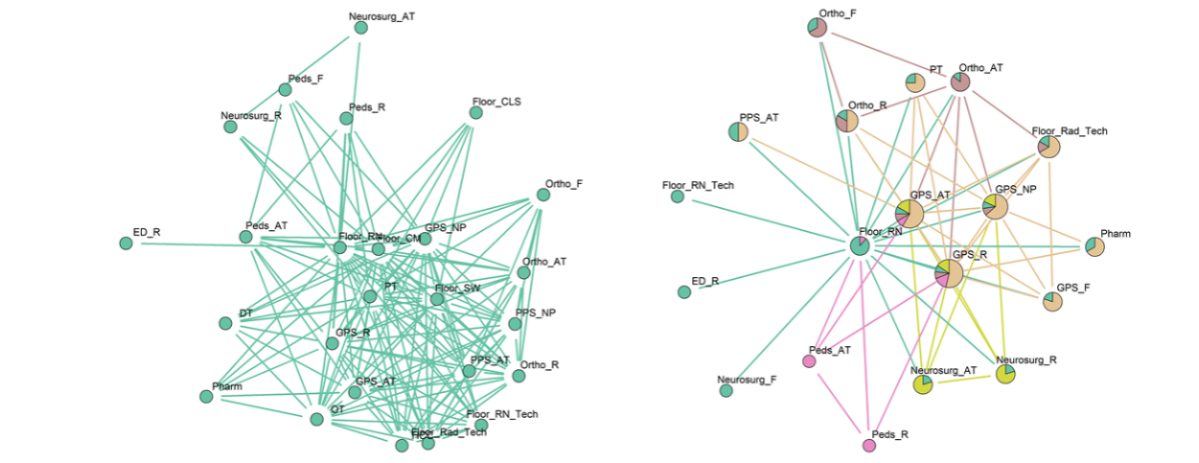
Collaborative care team pattern on the floor. Left: day; right: night. Only one community was identified in the day pattern while 5 communities (different colors) were identified in the night pattern.

Figure 4 shows the collaboration pattern on the floor during the day and at night visualized using the large graph layout [47]. The day pattern contained 24 nodes and 135 edges, whereas the night pattern contained 19 nodes and 55 edges. The bedside nurse was at the center of both patterns. Functional roles present in the day pattern but absent in the night pattern were home care coordinators, case managers, social workers, child life specialists, occupational therapy, and dietitians. The ED resident was present in the night pattern but not in the day pattern. One community was identified in the day pattern, whereas 5 overlapping communities were identified in the night pattern with the neurosurgery, orthopedic surgery, and pediatric services having separate communities and the general pediatric surgery-resident and general pediatric surgery attending belonging to all 5 communities.

### Collaboration Patterns of Care Teams in the PICU

**Figure 5:**
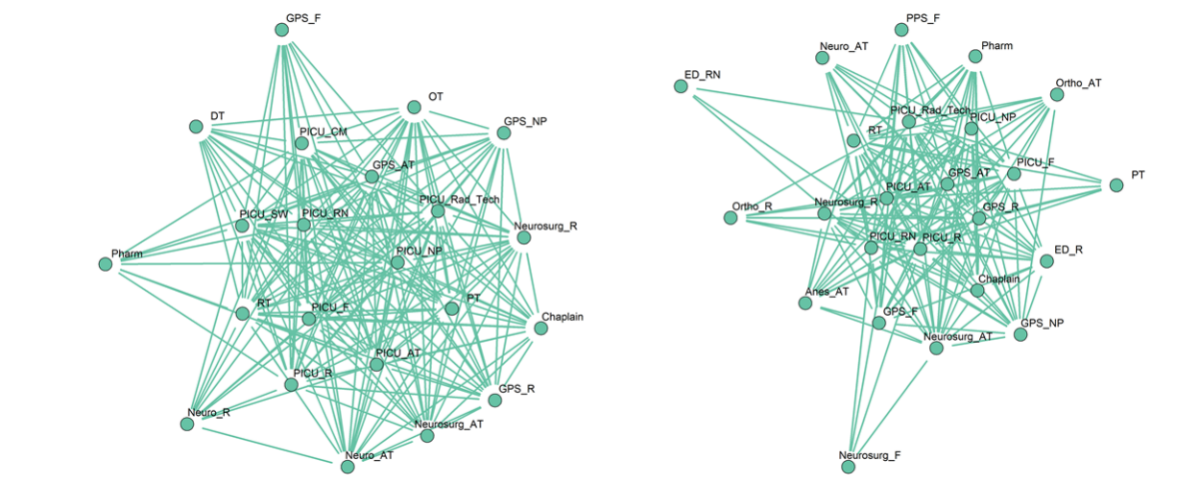
Collaborative care team patterns in the pediatric intensive care unit. Left: day; right: night.

Figure 5 shows the day and night collaborative care team pattern in the PICU visualized using the Frutchterman–Reingold layout algorithm [48]. The day pattern contained 30 nodes and 283 edges, whereas the night pattern contained 24 nodes and 175 edges. Both collaboration patterns had a large spherical core made up of functional roles from the PICU, general pediatric surgery, neurosurgery, and neurology services (day pattern only), and few *appendages* that include functional roles from the orthopedic surgery, ophthalmology and pediatric pain service. One community was identified in both the patterns. Functional roles present in the day pattern but absent in the night pattern were unit case managers, social workers, occupational therapists, and dietitians. Functional roles present in the night pattern but absent in the day pattern include the ED resident, ED nurse, orthopedic surgery team, and anesthesiology attending.

### Validation

In a previous study that used semistructured interviews with care professionals [45], we identified 56 roles involved in pediatric trauma care across all care locations. In this study, we identified a total of 110 functional roles and 58 frequent functional roles across all locations. Eight functional roles were identified in a previous study but not in this study. These roles were ED documenting nurses, charge nurses, emergency medical services personnel, security, family or caregiver, pediatric trauma manager, perfusionist, and in-hospital transport team. A total of 54 functional roles were identified in this study but not in a previous study. Most of these roles belonged to specialty service roles that were not frequently involved in patient care. Of the 58 frequent roles identified in this study, 15 (26%) were not identified in the prior study. These roles included dietitians, IDCs, home care coordinators, ophthalmology service, otolaryngology service, neurology service, pediatric pain service, and plastic surgery services.

A comparison between this study and the prior study showed that the locations of the functional roles they had in common mostly matched. For example, both studies confirmed that ED nurses, ED residents, and PICU nurses go to the floor, usually during patient transport. However, few differences exist. For example, in a previous study, it was revealed that the PICU attending, PICU fellow, and respiratory therapist responded to alpha traumas in the ED both during the day and at night, but this was not captured in this study.

The 6 HCPs who were interviewed for this study found the composition of the derived care teams to be largely accurate. However, they pointed out that some functional roles were not accurately captured. For example, PICU attendance, PICU fellows, and respiratory therapists were not identified in the ED collaborative care teams (Figure 3). This was attributed to the fact that PICU team members who responded to traumas rarely did any documentation while in the ED. They also pointed out that the team pattern for the PICU night did not capture the ED social worker who usually covers the PICU at night, and the floor care team patterns were missing functional roles from anesthesiology.

Regarding interactions among roles and communities that were identified, clinicians confirmed the general pediatric surgery coordinated care among specialist services and understood why they belonged to multiple communities. Clinicians explained why only 1 community was identified in the PICU, as concerted efforts have been made to improve coordination of care between the PICU and surgical services, and the PICU characteristically performed multidisciplinary rounds with other nonsurgical services and allied HCPs. However, clinicians acknowledged that collaboration with the orthopedic service, particularly in the PICU, can be further improved. Clinicians confirmed that the neurosurgery service was well integrated into the trauma team in the ED.

## Discussion

### Principal Findings

We compared diurnal differences in the composition and organization of collaborative care teams at 3 care locations in a level I pediatric trauma center using EHR data. Our study is unique in several ways. First, we introduced a heuristic for determining the threshold number of shared patient encounters for interaction between HCPs. The heuristic method allows a more objective approach to threshold selection. In 67% (4/6) of the scenarios, we obtained distinct elbow points, whereas in the other 33% (2/6) of the scenarios, we easily identified the appropriate threshold on closer examination. Second, we used an overlapping community detection algorithm that allowed a functional role to be part of multiple communities to reflect ad hoc clinical collaborations that clinicians form to address the unique needs of patients. In 33% (2/6; ED night and floor night) of the scenarios, we identified multiple overlapping communities suggestive of MTS, whereas in the other 67% (4/6), only a single community was identified. Third, we confirmed the presence of MTS using the EHR data. We also showed that the EHR data complemented interview data for identifying functional roles. Although interview data were especially helpful in identifying team members or roles who rarely document in the EHR, the EHR data enabled a more comprehensive and systematic analysis and identification of functional roles (56 functional roles identified with interviews vs 110 with EHR data).

There were 3 significant differences among patients who arrived during night shift compared with those who arrived during the day time. The patients who arrive at night tend to be older (median age 10 vs 7 years) and have penetrating injuries (11/141, 7.8% vs 5/272, 1.8%). This is likely related to prevailing epidemiological conditions and is consistent with what has been reported in the literature [49,50]. These patients also tended to arrive as transfers from other facilities (38/141, 27% vs 2/272, 0.7%). A higher percentage of transfers received at night reflects operational circumstances. Our pediatric trauma center is a level I trauma center that serves as a referral center for a large area. The decision to transfer patients is made by the originating facility, but several factors determine when patients physically arrive at our facility. First, the patients must be stabilized (to some extent) at the originating facility to ensure that they will survive transportation before departing the originating facility. Second, the level of staffing at the originating facility may influence transfer decisions such that patients who would be unsafe to manage at night when staffing is low are transferred to us after stabilization. Third, the distance of the originating facility and logistics of transportation can influence when transfer patients physically arrive at our facility.

There were some notable differences in the composition of event logs at various locations. The lower proportion of flow sheet activities in the ED is likely because of the relatively short time (usually <60 minutes) spent in the ED as compared with the entire hospital stay (usually days). The higher proportion of flow sheets and medication activities in the PICU compared with the ED and the floor reflects the intensive care provided in the PICU. The higher proportion of procedure orders in the ED compared with both the floor and PICU suggests the initiation and delivery of immediately necessary and likely lifesaving interventions.

Important differences were observed between the collaborative care teams in the ED during the day and at night. Compared with the day pattern, the night pattern had a better-defined core team made up of ED and general pediatric surgery personnel and involved more specialty services, which was reflective of the nature and severity of injury of patients presenting at night [8,51,52]. In addition, the neurosurgery team was part of both day and night patterns. However, in the night pattern, the neurosurgery team was part of the main community that included the core team and allied HCPs. This suggests that the neurosurgery team has a close relationship with the trauma team in the ED, which was confirmed by the interviewed clinicians. In addition, the collaborative care team in the ED at night included roles that did not exist in the day pattern. These roles include the IDC, a role that is responsible for uploading imaging data from transferring hospitals that do not use an interoperable EHR (which can be explained by the significantly higher number of trauma transfers arriving at night), and the PICU resident and PICU nurse, which suggested greater involvement at night, possibly to facilitate faster admission to the PICU). The orthopedic surgery attending and resident, and the ED resident and ED nurse were captured by the night pattern in the PICU but not during the day, which suggested greater involvement in PICU-related activities of trauma patients at night.

Compared with the day pattern, multi-team structures were more pronounced at night. Constituent specialty teams usually consisted of attending-resident pairs, except for the ophthalmology service, which consisted only of residents. Validation with clinicians confirmed that ophthalmology attending physicians do not take in-house night duty calls, given the seldom emergent nature of many ophthalmologic problems. Conspicuous multi-team structures reflected the presence of fewer ancillary support services that often serve as coordinators of care. In the ED, as ancillary support services were present at night, this may be reflective of the greater need of the patients received at night and the difficulty in coordination of care among the various services. On the floor, where ancillary support services are not present at night, this suggests that ancillary support staff play important roles in coordinating care and ensuring that various teams function as a unit. However, this was not the case in the PICU, where the night collaboration pattern was essentially similar to the day pattern despite the absence of ancillary support staff at night.

There are a number of reasons for the observed variations between day and night networks. As described, the level of staffing during the day was higher than that at night. During the day, more functional roles and support staff (care coordination, social work, etc) are present, and they participate in collaboration between teams. At night, some functional roles and nonessential staff are not available, which changes the dynamics of work and collaboration. In addition, more care activities (patient rounds, elective procedures, discharge planning, etc) occur during the day as opposed to nighttime. These activities create the need and opportunity for close collaboration compared with nighttime. Finally, there are likely differences in the manner of collaboration, for example, the use of non-EHR–based communications such as telephone and paging is more common during the night when team members tend to be more geographically dispersed, as opposed to during the day when they are geographically closer or physically working together.

In addition to organizational factors, methodological issues may also account for the variations. Only a single community was identified at all locations during the day. Although it is probable that the specialty teams actually do work very closely together during the day, it is likely that they do in a multi-team setup, which we did not identify by overlapping community detection. This may be owing to several reasons. The data may lack adequate *power* to detect overlapping communities during daytime. Certainly, smaller teams could be identified using cliques, which are unique subnetworks containing at least three nodes that are all connected to one another by edges. However, given the number of nodes involved, hundreds of overlapping cliques would be identified, which would be difficult to interpret. Another factor could be threshold selection. It is possible that by selecting different thresholds for day networks, we could identify overlapping communities. However, because the weights of the edges are considered by the community detection algorithm, such a sensitivity analysis was not required. Nevertheless, a narrow sensitivity analysis of the day networks using +/−1 the selected threshold did not show any major difference in terms of communities (Multimedia Appendices 1-3).

This study has several implications: the methodology can be adapted and used in other settings to identify and study MTS structures in an efficient manner. The methodology can also be adapted to study how MTS evolves over the care timeline of patients and identify areas in need of improvement. In-depth analysis of MTS across time, location, and team members using EHR metadata can provide insights to support management and operational decisions. For example, it can be used to derive insights into how HCPs and care teams organize themselves given the realities of *actual work*, rather than how they are supposed to organize according to protocols. Such insights can be used to inform staffing and team composition decisions, team training and development efforts, and complement efforts to improve collaboration and coordination to improve team-based health care delivery. In addition, the ability to compare temporal patterns in MTS dynamics based on EHR metadata enables assessment and evaluation of the impact of any quality improvement and intervention efforts aimed at improving MTS performance.

This study had several notable limitations. First, by only EHR data, we did not capture other important teamwork-related activities such as face-to-face and telephone conversations, which are a major part of clinical activities [53]. Second, we were less likely to capture functional roles that documented infrequently in the EHR. For example, we were unable to capture the PICU attending and PICU fellows in the ED patterns for both day and night, as these 2 roles rarely used the EHR for documentation while in the ED. We were also unable to capture several other HCPs, such as emergency medical services personnel, security, family and/or caregiver, pediatric trauma manager, perfusionist, and in-hospital transport team who rarely or never use the EHR for a trauma case, but are an integral part of the care team, as revealed by interview data. Other methods, such as in-depth interviews or direct observations, can be used to overcome these limitations. Third, our method for determining the functional roles was based on heuristics. Consequently, it is possible that not all possible roles were identified, and that some of the assigned functional roles were inaccurate. Nevertheless, as demonstrated, the methodology performs quite well; future EHR systems should be designed to support functional roles, which are the appropriate unit of clinical collaboration, rather than individuals; for example, clinical documentation could be primarily based on functional roles, but signed as individuals. Such systems have the potential to optimize collaborative work to deliver improved care and enable robust research using EHR data.

### Conclusions

We identified and described diurnal variations in MTS and collaborative care teams at various locations and stages of care, as well as various shift types in a pediatric trauma center using EHR data. We validated our results using qualitative data and showed that the derived structures can accurately represent reality. The methodology described can be adapted to study how MTSs evolve over time and across locations, and the insights can be used to support management and operational decisions.

## Appendix

Multimedia Appendix 1 Sensitivity analysis of the collaborative care team patterns for the emergency department day shift using +/−1 the selected threshold. At a lower threshold of 8, a minor overlapping community that included the orthopedic resident (Ortho_R) and imaging data coordinator (IDC) as additional members was identified.

Multimedia Appendix 2 Sensitivity analysis of the collaborative care team patterns for the floor day shift using +/−1 the selected threshold. At a threshold of 10, functional roles with single connection to the floor nurse were identified. However, these functional roles were removed as the threshold is increased with no functional role having a single connection at a threshold of 12.

Multimedia Appendix 3 Sensitivity analysis of the collaborative care team patterns for the pediatric intensive care unit day shift using +/−1 the selected threshold. At a higher threshold of 16, a minor community including the neurology resident as the only additional member was identified.

## Abbreviations

ADT: admission, discharge, and transfer
ED: emergency department
EHR: electronic health record
HCP: health care professional
IDC: imaging data coordinator
LOWESS: Locally Weighted Scatterplot Smoothing
MTS: multi-team system
PICU: pediatric intensive care unit
