# Supplementary figures and images for: Examining Diurnal Differences in Multidisciplinary Care Teams at a Pediatric Trauma Center Using Electronic Health Record Data: Social Network Analysis
Source: J Med Internet Res. 2022 Feb 4;24(2):e30351. doi: 10.2196/30351 (PMC8857698; doi:10.2196/30351)

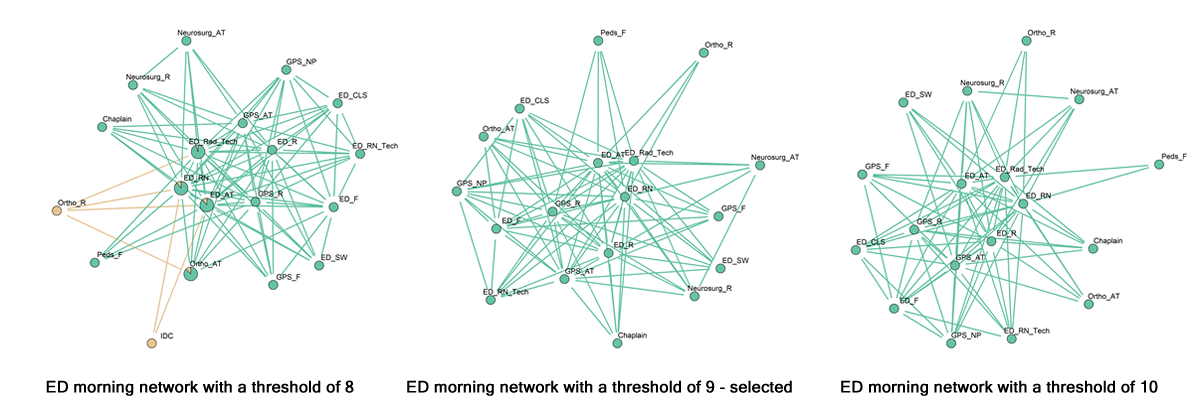

Supplement: Multimedia Appendix 1 [file jmir_v24i2e30351_app1.png]

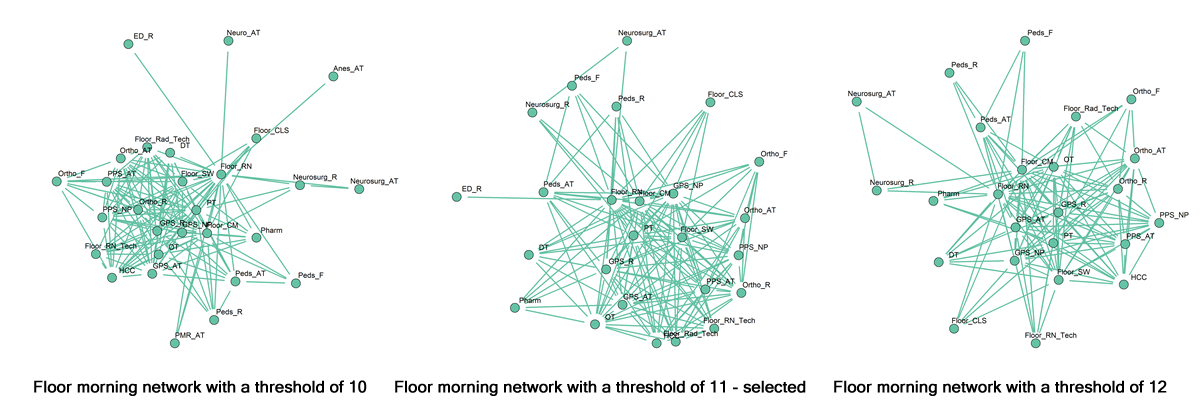

Supplement: Multimedia Appendix 2 [file jmir_v24i2e30351_app2.png]

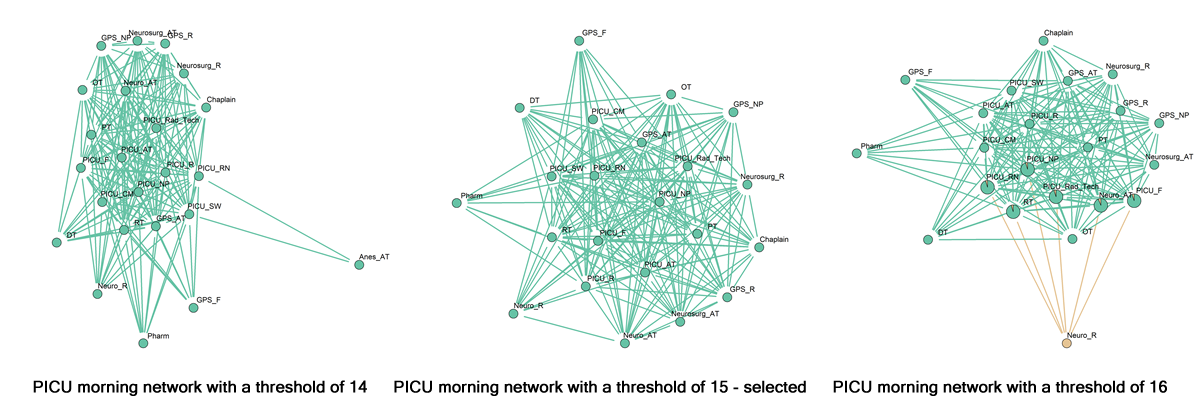

Supplement: Multimedia Appendix 3 [file jmir_v24i2e30351_app3.png]
